# Development of a predictive model for post-surgical chronic pain: a retrospective analysis of calcaneal fracture patients

**DOI:** 10.1186/s12891-025-08428-y

**Published:** 2025-02-19

**Authors:** Shibo Zhai, Shuo Zhang, Xinzhe Ma, Yongchao Gong, Zhiyong Hou, Wei Chen, Lijie Ma

**Affiliations:** Department of Orthopaedic Surgery, Hebei Medical University Third Hospital, Shijiazhuang, 050051 China

**Keywords:** Calcaneal fracture, Chronic pain, Risk prediction, Nomogram, Retrospective study

## Abstract

**Background:**

Chronic pain following calcaneal fracture surgery substantially diminishes patients' quality of life, yet effective risk prediction tools remain scarce. This study aimed to develop and validate a reliable predictive model for assessing the risk of chronic pain development after calcaneal fracture surgery.

**Methods:**

This retrospective analysis examined 398 patients who underwent calcaneal fracture surgery between January 2022 and July 2023. Patients were randomly allocated into model development (*n* = 280) and validation (*n* = 118) cohorts. Independent risk factors were identified through multivariate logistic regression analysis to construct the prediction model. Model performance was evaluated using the area under the receiver operating characteristic (ROC) curve, calibration plots, and decision curve analysis.

**Results:**

The study identified several independent predictors of postoperative chronic pain: body mass index (BMI) (OR:1.10, 95%CI:1.02–1.19, *P* = 0.014), operative duration (OR:1.01, 95%CI:1.00–1.02, *P* = 0.019), surgical approach, and Böhler angle (OR:1.03, 95%CI:1.00–1.06, *P* = 0.025). The predictive model demonstrated good discriminative ability in both development and validation cohorts, with AUC values of 0.691 (95%CI:0.63–0.75) and 0.655 (95%CI:0.56–0.77), respectively. Calibration plots showed strong agreement between predicted probabilities and observed outcomes.

**Conclusion:**

Our newly developed predictive model, incorporating BMI, operative duration, surgical approach, and Böhler angle, effectively predicts the risk of chronic pain following calcaneal fracture surgery. This tool provides valuable guidance for clinicians in conducting individualized risk assessments and implementing targeted preventive strategies.

## Introduction

Calcaneal fractures represent a significant challenge in orthopedic trauma, accounting for approximately 2% of all fractures and 60–65% of all tarsal fractures in adults [1–3]. These injuries are predominantly caused by high-energy trauma, such as falls from height or motor vehicle accidents, with 80–90% of affected patients being young to middle-aged males [4]. While surgical intervention is the preferred treatment for many calcaneal fractures, achieving favorable outcomes in many cases, the emergence of chronic postoperative pain has become a significant challenge in clinical management. Chronic postoperative pain significantly impacts patients' quality of life and imposes substantial psychological and economic burdens [5].

Despite numerous studies proposing potential risk factors for chronic pain, a significant gap remains between their validity and clinical utility. Predictive modeling offers considerable value in clinical applications by effectively identifying high-risk populations and assisting clinicians in early diagnosis and personalized intervention. However, there is a paucity of comprehensive analysis and prediction models for chronic pain risk following calcaneal fracture surgery that fully integrates and visualize multiple risk factors.

This study aims to develop a predictive model for chronic pain risk following calcaneal fracture surgery in adults and to systematically analyze the various risk factors influencing postoperative chronic pain. By identifying high-risk patients early and implementing appropriate interventions, we propose a personalized treatment strategy tailored to each individual's risk profile. Ultimately, this approach aims to enhance postoperative recovery, mitigate pain, and improve the overall quality of life for patients undergoing calcaneal fracture surgery.

## Materials and methods

### Study design and participants

This retrospective cohort study was conducted at the Third Hospital of Hebei Medical University. Patients who underwent surgical treatment for calcaneal fractures between January 2022 and July 2023 were eligible for inclusion. The study protocol was approved by the Ethics Committee of the Third Hospital of Hebei Medical University (approval number: ke2024-097–1), and written informed consent was obtained from all participants.

#### Inclusion criteria

1. Age ≥ 18 years
2. Clinical and radiological diagnosis of calcaneal fracture
3. Underwent surgical treatment for calcaneal fracture
4. Minimum follow-up duration of 6 months

#### Exclusion criteria

1. Preoperative infection in any part of the body
2. Pathological calcaneal fractures
3. History of malignant tumors or immune dysfunction
4. Presence of local calcaneal deformity
5. Contraindications to surgery
6. Malunion of calcaneal fractures
7. Bilateral calcaneal fractures

### Data collection

Demographic and clinical data were collected from electronic medical records and imaging systems. Variables included patient age, gender, body mass index (BMI), fracture characteristics (site, Sanders CT classification) [6], surgical details (method, duration, intraoperative bleeding), and postoperative factors (complications, implant removal). Radiographic measurements included Böhler angle, Gissane angle, calcaneal length, and width. A total of 22 variables were recorded for each patient.

### Outcome measure

The primary outcome was the presence of chronic postoperative pain, defined according to the International Association for the Study of Pain (IASP) criteria for Chronic Post-Surgical Pain (CPSP): pain persisting at the surgical site for at least 3 months postoperatively [7].Pain severity was assessed using the Visual Analog Scale (VAS), where 0 indicates no pain, 1–3 mild pain, 4–6 moderate pain, and 7–10 severe pain. Patients with a VAS score ≥ 1 were categorized into the pain group, while those with a score of 0 were placed in the non-pain group. Most patients had completed functional training at 12 months; this study mainly used the VAS score at 12 months as the evaluation index. The score data were obtained by telephone follow-up.

### Statistical analysis

Statistical analyses were performed using SPSS version 26.0 (IBM Corp., Armonk, NY, USA) and R version 4.4.0 (R Foundation for Statistical Computing, Vienna, Austria). The normality of continuous variables was assessed using the Kolmogorov–Smirnov test. Normally distributed data were expressed as mean ± standard deviation (SD), while non-normally distributed data were presented as median (interquartile range). Categorical variables were expressed as frequencies and percentages.

Comparisons between the pain and non-pain groups were made using independent samples t-test or Mann–Whitney U test for continuous variables, and chi-square test or Fisher's exact test for categorical variables, as appropriate. Variables with *P* < 0.2 in univariate analysis were included in the multivariate logistic regression analysis to identify independent risk factors for chronic pain after calcaneal fracture surgery.

A nomogram was constructed based on the results of the multivariate logistic regression analysis. The predictive performance of the nomogram was evaluated using the area under the receiver operating characteristic (ROC) curve (AUC). The model's calibration was assessed using calibration curves and the Hosmer–Lemeshow goodness-of-fit test. A two-tailed *P* value < 0.05 was considered statistically significant for all analyses.

### Quality control

To ensure data quality and consistency, all relevant clinical information and imaging data were collected, organized, and analyzed by qualified radiologists and orthopedic surgeons who received standardized training before the study. The data collection and analysis processes were supervised and randomly audited by a chief orthopedic surgeon and a chief radiologist.

## Results

### Comparison of general information between the pain and non-pain groups

A total of 398 patients were enrolled and randomized in a 7:3 ratio into training (*n* = 280) and validation (*n* = 118) sets. In the training cohort, male patients predominated (*n* = 254, 90.7%) compared to females (*n* = 26, 9.3%). The gender distribution showed no significant difference between the pain-free group (males: 87.1%, females: 12.9%) and pain group (males: 92.7%, females: 7.3%) (*P* = 0.181). However, significant between-group differences were observed in surgical procedure selection (*P* = 0.015) and body mass index (BMI) (*P* = 0.020). Specifically, screw fixation was performed in 41 cases (14.6%) and plate fixation in 167 cases (59.6%), with differing distributions between the pain-free (screw: 14.9%, plate: 69.3%) and pain groups (screw: 14.5%, plate: 54.2%). Mean BMI values were notably higher in the pain group (26.52 ± 3.55 kg/m^2^) compared to the pain-free group (24.91 ± 3.38 kg/m^2^). These findings suggest that surgical approach and BMI may serve as potential predictive factors for post-operative pain outcomes.

### Results of univariate analysis of chronic pain

Three factors significantly associated with the occurrence of chronic pain were identified by univariate analysis. First, an increase in BMI (body mass index) was positively associated with the occurrence of pain (OR: 1.09, 95%CI: 1.01–1.17, *P* = 0.022). Secondly, the difference in surgical procedure (Kirschner wire fixation) was potentially associated with pain (OR: 2.02, 95%CI: 0.87–4.73, *P* = 0.103), although this result did not reach statistical significance (Table 1). Finally, a longer duration of surgery was associated with an increased risk of pain occurrence (OR: 1.01, 95%CI: 1.00–1.02, *P* = 0.028), suggesting that a longer duration of surgery may increase the risk of postoperative pain. These results provide important clues to our understanding of the factors influencing the development of chronic pain (Table 2).

**Table 1:** Comparison of general conditions of patients in two groups

Variable	Category	Total Cases (*n* = 280)	Pain-Free Group (*n* = 101)	Pain Group (*n* = 179)	*P* Value
Gender	Male	254 (90.7%)	88 (87.1%)	166 (92.7%)	0.181
	Female	26 (9.3%)	13 (12.9%)	13 (7.3%)	
Smoking History	No	165 (58.9%)	66 (65.3%)	99 (55.3%)	0.130
	Yes	115 (41.1%)	35 (34.7%)	80 (44.7%)	
Alcohol Consumption	No	164 (58.6%)	59 (58.4%)	105 (58.7%)	1.000
	Yes	116 (41.4%)	42 (41.6%)	74 (41.3%)	
Hypertension History	No	246 (87.9%)	91 (90.1%)	155 (86.6%)	0.501
	Yes	34 (12.1%)	10 (9.9%)	24 (13.4%)	
Diabetes History	No	271 (96.8%)	99 (98.0%)	172 (96.1%)	0.598
	Yes	9 (3.2%)	2 (2.0%)	7 (3.9%)	
Surgical Procedure	Screw Fixation	41 (14.6%)	15 (14.9%)	26 (14.5%)	0.015
	Plate Fixation	167 (59.6%)	70 (69.3%)	97 (54.2%)	
BMI (kg/m^2^)	-	25.56 ± 3.51	24.91 ± 3.38	25.92 ± 3.55	0.020
Surgery Time (min)	-	75 (60–90)	70 (60–85)	75 (60–90)	0.094
Intraoperative Blood Loss (ml)	-	95 (50–100)	100 (60–100)	80 (50–100)	0.171
Böhler's Angle (°)	-	28.3 (21.3–34.8)	28.2 (20.6–33.8)	28.4 (22.0–35.2)	0.266
Gissane's Angle (°)	-	114.8 (107.8–122.1)	114.8 (108.9–120.6)	114.8 (107.4–122.4)	0.941
Calcaneal Length (cm)	-	7.72 ± 0.59	7.72 ± 0.62	7.72 ± 0.57	0.991
Calcaneal Height (cm)	-	4.96 ± 0.50	4.94 ± 0.45	4.96 ± 0.53	0.753
Calcaneal Width (cm)	-	5.53 ± 0.79	5.42 ± 0.70	5.60 ± 0.84	0.073

**Table 2:** Results of one-way logistic regression

Variable	Category	OR (95% CI)	*P*-value
Gender	Female	0.53 (0.23–1.20)	0.125
Age	-	1.01 (0.99–1.03)	0.319
BMI	-	1.09 (1.01–1.17)	0.022
Implant removal	Yes	0.62 (0.38–1.02)	0.060
Laterality (Right side)	Right side	0.74 (0.43–1.25)	0.261
Laterality (Bilateral)	Bilateral	1.12 (0.54–2.39)	0.761
Smoking history	Yes	1.52 (0.92–2.54)	0.102
Alcohol history	Yes	0.99 (0.60–1.63)	0.968
Hypertension history	Yes	1.41 (0.66–3.21)	0.390
Diabetes history	Yes	2.01 (0.48–13.7)	0.388
Surgical procedure (Kirschner wire fixation)	Kirschner wire fixation	2.02 (0.87–4.73)	0.103
Surgical procedure (Plate fixation)	Plate fixation	0.80 (0.39–1.60)	0.534
Postoperative complication (No deep vein thrombosis)	No deep vein thrombosis	0.88 (0.32–2.19)	0.786
Subtalar joint fusion	Yes	0.55 (0.27–1.11)	0.090
Osteoarthritis	Yes	1.01 (0.50–2.09)	0.981
Radiographic staging (Type 2)	Type 2	1.19 (0.67–2.08)	0.542
CT classification (Type 1)	Type 1	0.87 (0.23–3.28)	0.842
CT classification (Type 2)	Type 2	1.09 (0.38–2.99)	0.867
CT classification (Type 3)	Type 3	1.08 (0.37–3.02)	0.885
CT classification (Type 4)	Type 4	1.68 (0.51–5.45)	0.388
Surgery duration	-	1.01 (1.00–1.02)	0.028
Intraoperative blood loss	-	1.00 (1.00–1.00)	0.887
Böhler angle	-	1.02 (1.00–1.04)	0.122
Gissane's angle	-	1.00 (0.98–1.02)	0.726
Calcaneal length	-	1.00 (0.66–1.51)	0.991
Calcaneal height	-	1.08 (0.67–1.79)	0.752
Calcaneal width	-	1.34 (0.98–1.87)	0.075

### Results of multifactorial logistic regression analysis of chronic pain

Pain occurrence was used as the dependent variable, and variables with a *P* value of less than 0.2 in the univariate analysis were included in the model. Factors independently associated with chronic pain occurrence were identified by combining the results of the Hosmer–Lemeshow test and multifactorial analysis. The results showed that BMI, duration of surgery, surgical approach, and Böhler angle were independent predictors of the development of chronic pain after calcaneal fracture surgery (Tables 3 and 4).

**Table 3:** Assignment of variables for multifactor logistic regression analysis

Variable	Assignment Description
Dependent variable	0 = No pain, 1 = Pain
Gender	1 = Male, 2 = Female
BMI	Continuous variable
Surgery duration	Continuous variable
Surgical procedure	1 = Screws, 2 = Kirschner wires, 3 = Incised steel plate
Böhler angle	Continuous variable

**Table 4:** Results of multifactor logistic regression

Variable	B	Wald	*P*-value	OR	95%CI (Lower)	95%CI (Upper)
Constant	−3.52	8.85	0.003	-	-	-
Gender (Male vs Female)	−0.67	2.38	0.123	0.51	0.22	1.20
BMI	0.10	6.05	0.014	1.10	1.02	1.19
Surgery duration	0.01	5.53	0.019	1.01	1.00	1.02
Surgical procedure1		10.09	0.006	-	-	-
Surgical procedure2	0.86	3.63	0.057	2.37	0.98	5.75
Surgical procedure3	−0.23	0.39	0.534	0.79	0.38	1.65
Böhler angle	0.03	5.00	0.025	1.03	1.00	1.06

### Construction of a predictive model for postoperative chronic pain column charts

**Fig. 1:**
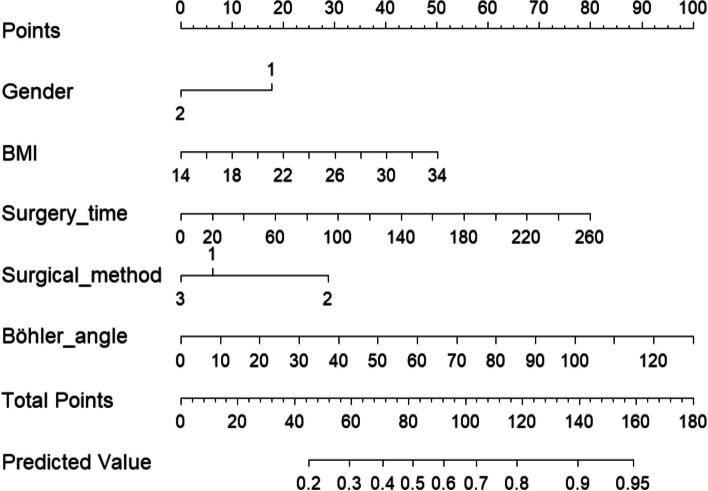
Postoperative chronic pain column of calcaneal fracture

**Fig. 2:**
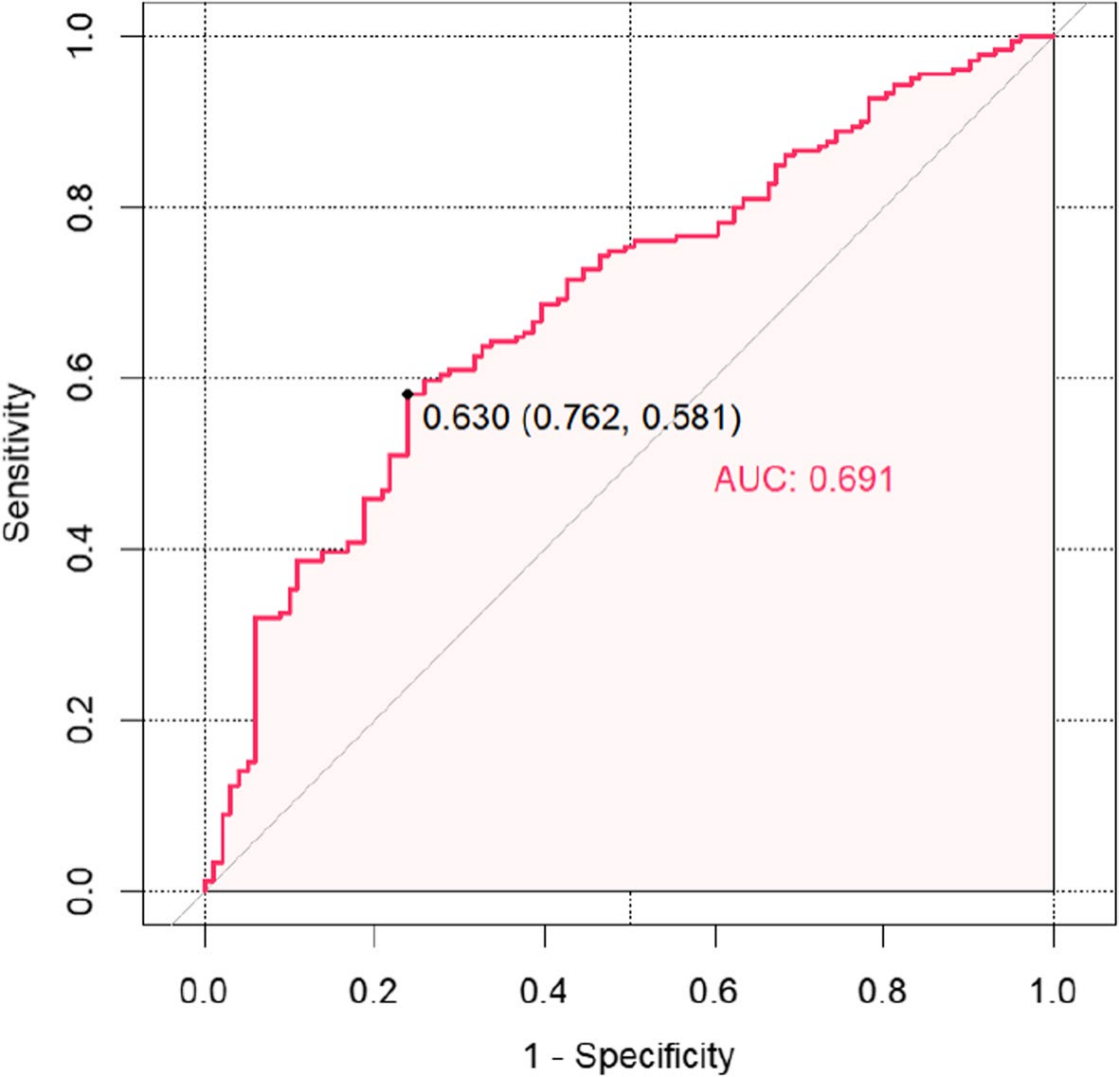
ROC graph for training set

**Fig. 3:**
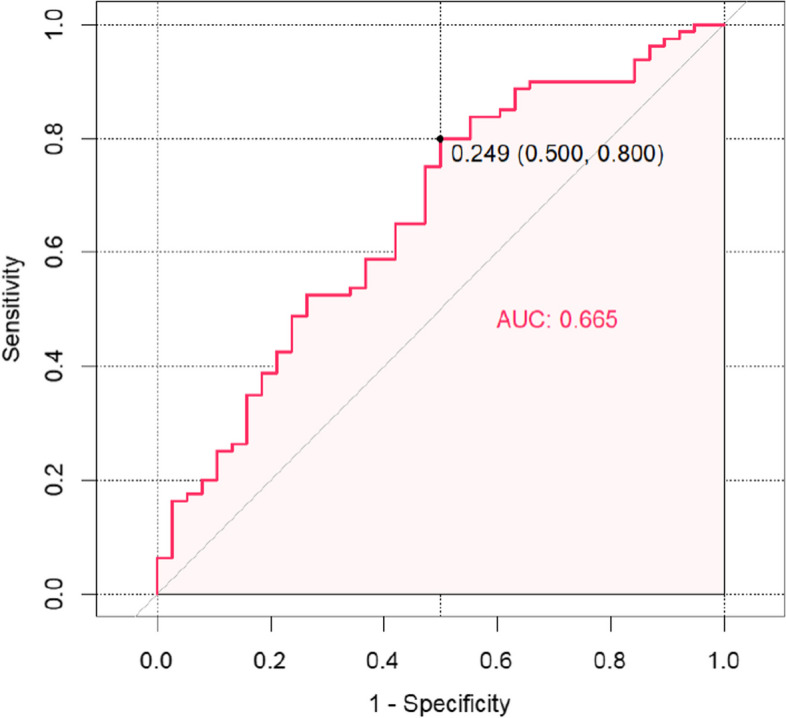
ROC graph for validation set

Based on the results of the multifactorial analysis, a column-line graphical model was constructed to predict chronic pain after calcaneal fracture surgery, with different values of each risk factor corresponding to the probability of pain occurrence (see Fig. 1). These risk factors included gender, BMI, duration of surgery, surgical approach, and Böhler angle. Using the risk of postoperative chronic pain in patients with calcaneal fracture obtained from the column-line diagram model as the test variable, we recorded the actual occurrence of postoperative pain as the state variable. We plotted the ROC curve of the column-line diagram model to predict postoperative chronic pain in calcaneal fracture. The AUC of the ROC curve for the training set was 0.691 (95% CI: 0.63 to 0.75) (Fig. 2). The AUC of the ROC curve for the validation set was 0.655 (95% CI: 0.56 to 0.77) (Fig. 3). The training and validation sets indicate that this prediction model has good prediction efficacy.

### Model validation

**Fig. 4:**
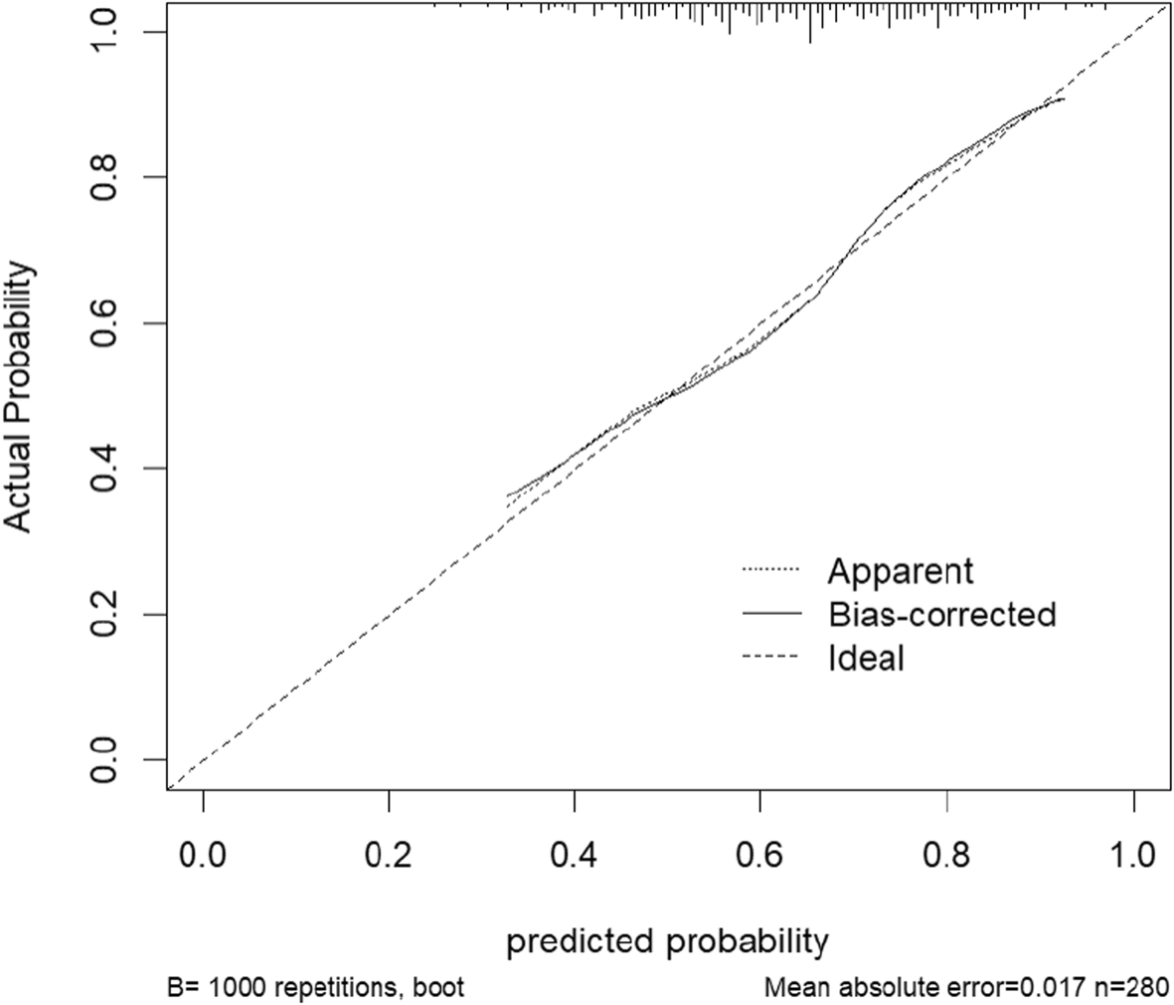
Training set calibration curve

**Fig. 5:**
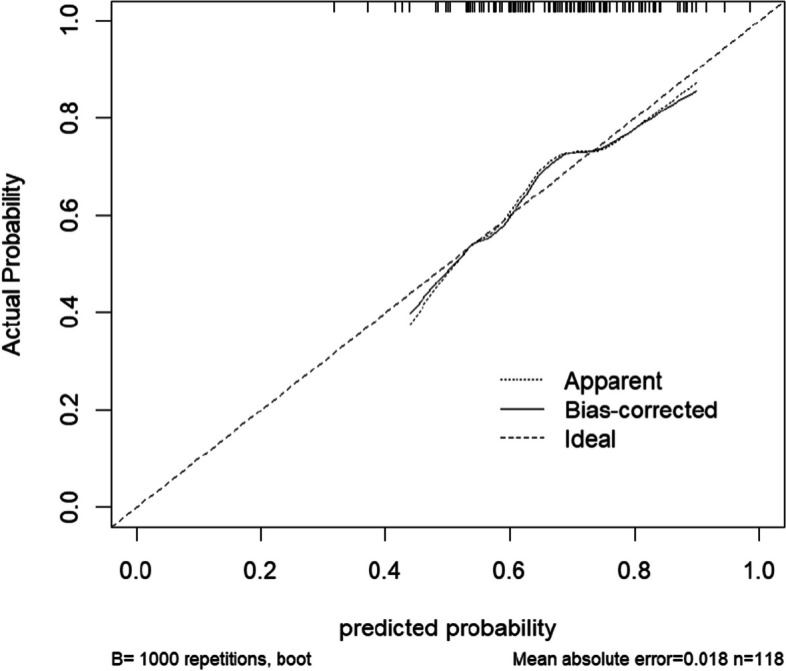
Validation set calibration curve

To further validate the accuracy of the model, a calibration curve for the model was plotted and a Hosmer–Lemeshow goodness-of-fit test was implemented. In the calibration plot, the calibration curve has the predicted probability on the X-axis and the actual probability on the Y-axis. The results show that the calibration curves of the training and validation sets fit the ideal curve well (Figs. 4, 5), and the Hosmer–Lemeshow test for the training set shows ${x}^{2}$= 6.946, *P* = 0.542, and the validation set Hosmer–Lemeshow result shows ${x}^{2}$ =2.813, *P* = 0.946. indicating that the model has good calibration ability.

### Evaluation of clinical utility

**Fig. 6:**
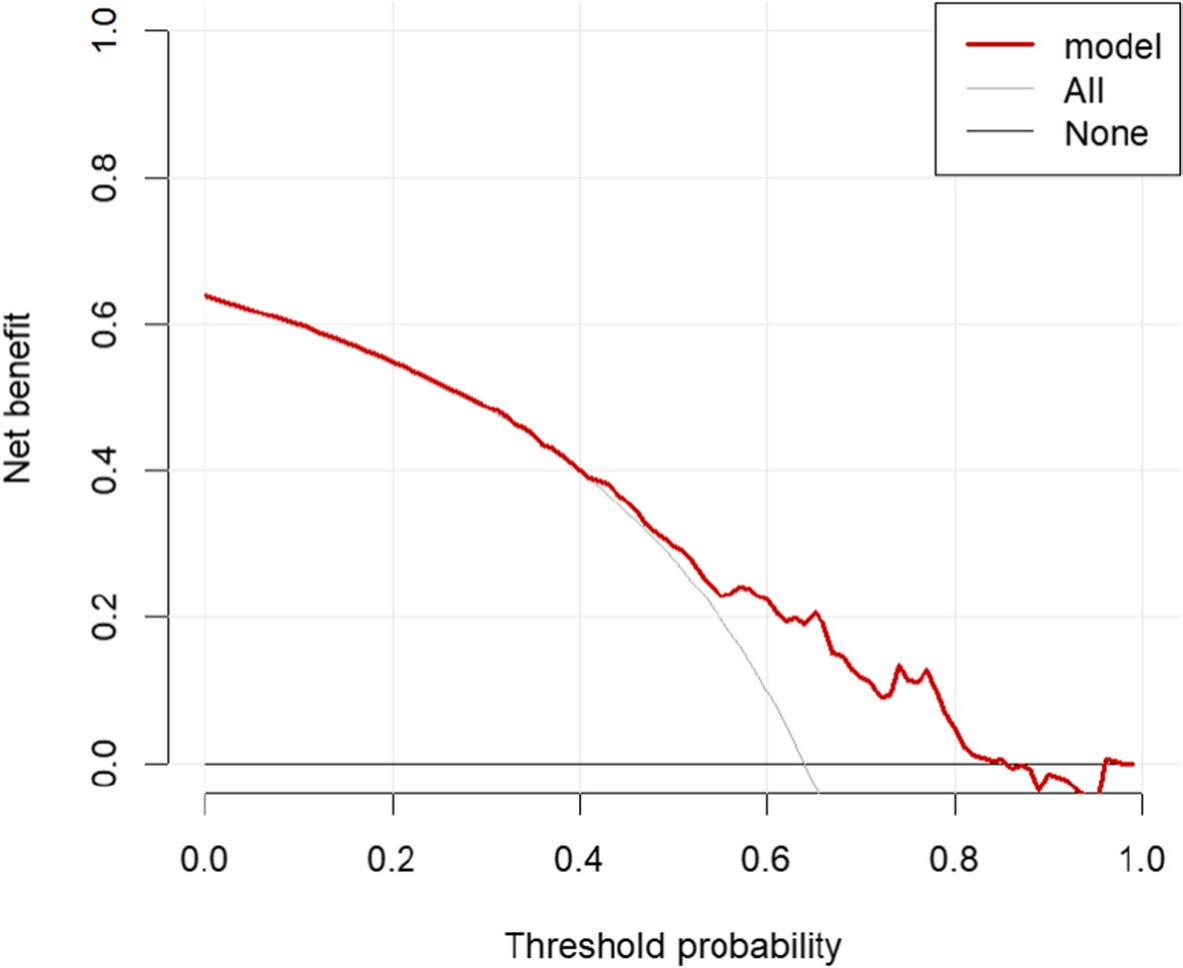
Training set decision curve

**Fig. 7:**
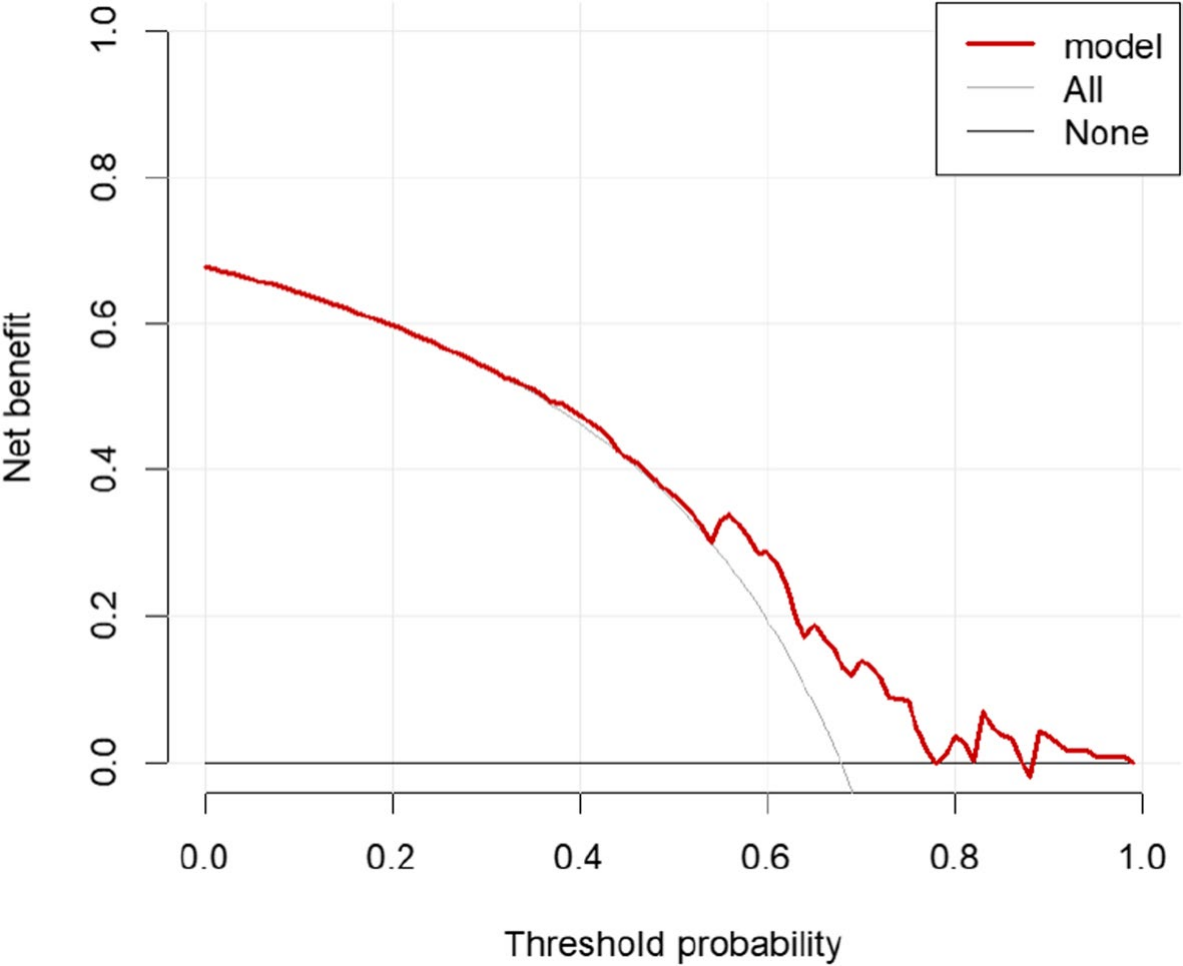
Validation set decision curve

Decision curve analysis (DCA) is often used to evaluate the clinical utility of predictive models [8]. The vertical coordinate represents the net benefit, which is obtained by subtracting the predictive model from the true probability of occurrence; the horizontal coordinate represents the threshold probability. The results of the training set showed a prediction range of 0.42–0.85, and the results of the validation set showed a prediction range of 0.50–0.90 (Figs. 6, 7), the prediction of the model is more accurate, and the patients can be benefited from the clinical diagnosis and treatment.

## Discussion

Currently, the incidence of calcaneal fracture in China is 11.5/100000 [9], of which 60%−80% of calcaneal fractures involve the articular surface. Intra-articular fractures of the calcaneal bone are a serious injury, Even with surgical treatment, patients often take a long time to return to full-time work [10]. Tøien, K et al. found that patients with calcaneal fractures tended to have lower health quality of life scores after surgery, particularly in terms of pain and activities of daily living [11]. Therefore reducing patients' postoperative pain becomes especially important. This paper analyzes and summarizes the causes of postoperative pain in follow-up cases at the Third Hospital of Hebei Medical University to provide a theoretical reference for clinical rehabilitation after calcaneal fracture and to reduce the incidence of chronic postoperative pain after calcaneal fracture. Simske et al. concluded that age, gender, smoking status, mechanism of injury, type of fracture, and postoperative infections were not associated with the outcome scores, which is partially consistent with the present study [12]. In this study, factors such as BMI, duration of surgery, surgical approach, and Böhler angle were found to be associated with postoperative chronic pain in patients.

Body mass index (BMI) does play a crucial role in patients' postoperative recovery. 780 patients analyzed by Majedi et al. found that chronic pain was associated with patients' BMI [13]; Lu et al. found that incisional complications were significantly increased with a BMI ≥ 26.4 kg/m^2^, which may be related to obesity leading to larger surgical incisions and more operating This may be related to the fact that obesity leads to larger surgical incisions and more operation time and more soft tissue injuries [14]. This may be related to the fact that obesity leads to larger surgical incisions more operation time and more soft tissue damage; this paper also concluded that patients with a high BMI are at a higher risk of developing postoperative chronic pain.

Surgical time is a key variable in the surgical procedure. The duration of surgery not only reflects the complexity of the surgical procedure but more importantly indicates the complexity of the fracture. While CT classification can reflect fracture complexity to some extent, the actual conditions found during surgery are often more complicated. Extended operation time may indicate more severe fracture displacement and more articular surface fragmentation, which may affect postoperative pain occurrence. No correlation was found between the type of fracture and chronic postoperative pain, which may be attributed to the small sample size of the study. As the duration of the surgery increases, the risk of intraoperative soft tissue damage and postoperative infection correspondingly rises. The study by Ding et al. concluded that surgical time is associated with the incidence of wound complications after incisional reduction and internal fixation of calcaneal fractures with plates [15]. Desborough's article highlights the fact that prolonged surgical time leads to more tissue damage and higher levels of inflammatory factors, which in turn leads to a stronger inflammatory response and postoperative pain [16]. The article also emphasizes that the length of surgery not only reflects the complexity of the surgical procedure but may also affect postoperative tissue healing and patient recovery. Another point is that since the tourniquet was only used in patients with open reduction and internal fixation, and the tourniquet was not used in percutaneous Kirschner wire and percutaneous screw fixation surgery, this study did not use the tourniquet use time as an analysis factor, but it is undeniable that use of a tourniquet during surgery was also one important factor that might affect the final postop outcomes. The longer it was applied, the longer ischemic time would be which would lead to more tissue damage postop. Prolonged ischemic time may lead to more tissue damage, which in turn affects postoperative pain. This is worth further exploration in future studies.

There is a lack of broad consensus on the treatment strategy for calcaneal fractures in terms of whether to choose conservative treatment, surgical treatment, or the best surgical approach [17, 18]. Complex calcaneal fractures often result in persistent postoperative pain, calcaneal deformity, and delayed healing [19–21]. This study found that compared to percutaneous cannulated screw fixation and open reduction and internal fixation, percutaneous Kirschner wire fixation is more likely to result in postoperative pain. The higher incidence of chronic postoperative pain in the percutaneous Kirschner wire fixation group may be attributed to the following factors: First, anatomical reduction of the articular surface is critical for prognosis. Percutaneous procedures relying solely on fluoroscopic guidance may fail to achieve optimal reduction, particularly when assessing and reducing the posterior articular surface. Therefore, the choice of surgical approach should be based on a detailed preoperative CT evaluation. For cases with severe posterior articular displacement or comminution, open reduction may be more effective in achieving better outcomes. Second, the percutaneous Kirschner wire fixation technique has a learning curve, and differences in surgical experience may also affect outcomes. Wang Qiuyuan et al. analyzed 902 patients with displaced intra-articular calcaneal fractures and found that hollow screw fixation had a similar postoperative function as plate fixation, which was satisfactory. Hollow screw fixation was superior to plate fixation in terms of quality reduction, time efficiency, and wound complications [22]. Many studies have also proposed screw-nailing techniques with favorable imaging and functional outcomes [23–25]. Our previous research also confirmed that percutaneous cannulated screw fixation offers advantages such as minimal trauma, reduced soft tissue damage, and fewer postoperative complications. In summary, percutaneous cannulated screw fixation may be more advantageous in the treatment of calcaneal fractures.

Böhler angle horn was introduced in 1931 as an important anatomical indicator of calcaneal bone [26]. There is a large body of evidence that patients benefit better from restoring the Böhler angle to near normal after a calcaneal fracture [27–29]. Regardless of treatment, patients with significantly reduced Böhler angle perform worse on the VAS and SF-36 scores at 2 years after calcaneal fracture surgery. Buckley et al. analyzed that surgical restoration of the Böhler angle significantly improved functional outcomes and reduced pain.Buckley et al. Randomized controlled trials further support the benefits of surgical restoration of the Böhler angle in reducing long-term pain and improving functional outcomes [30]. The Böhler angle has been shown to reduce pain and improve functional outcomes. In summary, a reduction in the Böhler angle does have a significant association with increased hindfoot pressure, cartilage wear, arthritis, and chronic pain. The above literature provides solid support for this relationship. Orthopedic surgeons should try to restore the Bohler’s angle when managing calcaneal fractures to ensure that patients have a better prognosis and less chronic pain after surgery. Böhler and Gissane angles are important parameters for evaluating calcaneal anatomical reduction, but these two-dimensional measurements do have limitations. Postoperative CT scans can more accurately assess the quality of articular surface anatomical reduction. In clinical practice, detailed preoperative CT evaluation is crucial for surgical approach selection, especially for posterior facet assessment.

The chronic pain risk prediction model constructed in this study, evaluated using ROC curves and the Hosmer–Lemeshow goodness-of-fit test, demonstrated high predictive efficacy and good calibration ability. This result not only validates the reliability and practicality of the model but also provides an actionable tool for clinical application. Compared with traditional single-risk-factor assessment methods, this model integrates multiple clinical variables, offering an important reference for clinicians in developing individualized treatment plans.

This study has several limitations, including the failure to collect data on patient's psychological status (such as anxiety and depression scale scores), the lack of preoperative VAS scores as a baseline reference, and the failure to record the postoperative rehabilitation program in detail, which may affect the occurrence and assessment of chronic pain. In addition, the retrospective design of the study introduced potential recall bias and limited causal inference, the single-center nature, and relatively small sample size may affect the generalizability of the study results, and the short study period may not capture the long-term pain trajectory. Therefore, future studies should address these limitations through prospective, multi-center, larger sample sizes, more included factors, and longer follow-up studies to more accurately assess the occurrence of chronic pain and its influencing factors.

In conclusion, this study found that BMI, operative time, surgical method, and Böhler angle were associated with chronic pain after calcaneal fracture surgery. Future research should focus on external validation of the model, exploration of additional risk factors (including psychological variables), and development of targeted interventions for high-risk patients. Ultimately, these efforts aim to enhance postoperative pain management and increase patient satisfaction in calcaneal fracture treatment.

## Data Availability

Availability of data and materials The datasets generated and/or analyzed during the current study are available from the corresponding author upon reasonable request.
